# Supplementary material for: ClinGen API platform for classification of human genetic variants
Source: Cell Genom. 2026 Apr 8;6(4):101211. doi: 10.1016/j.xgen.2026.101211 (PMC13069835; doi:10.1016/j.xgen.2026.101211)
Supplement: Document S1. Figure S1 and Table S1 [file mmc1.pdf]

Cell Genomics, Volume *6*

Supplemental information

ClinGen API platform

for classification of human genetic variants

Neethu Shah, Tierra Farris, Arturo Alejandro Zuniga, Andrew R. Jackson, Jessie Arce, Kevin Riehle, Christine G. Preston, Mark E. Mandell, Bryan Wulf, Gloria Cheung, Keyang Yu, Deborah I. Ritter, Dubravka Jevtic, Miroslav Milinkov, Novak Martinovic, Nevena Vucinic, Aleksandar Mihajlovic, Alan F. Rubin, Melissa S. Cline, Marina Distefano, Malachi Griffith, Obi L. Griffith, Matt W. Wright, Teri E. Klein, Sharon E. Plon, and Aleksandar Milosavljevic

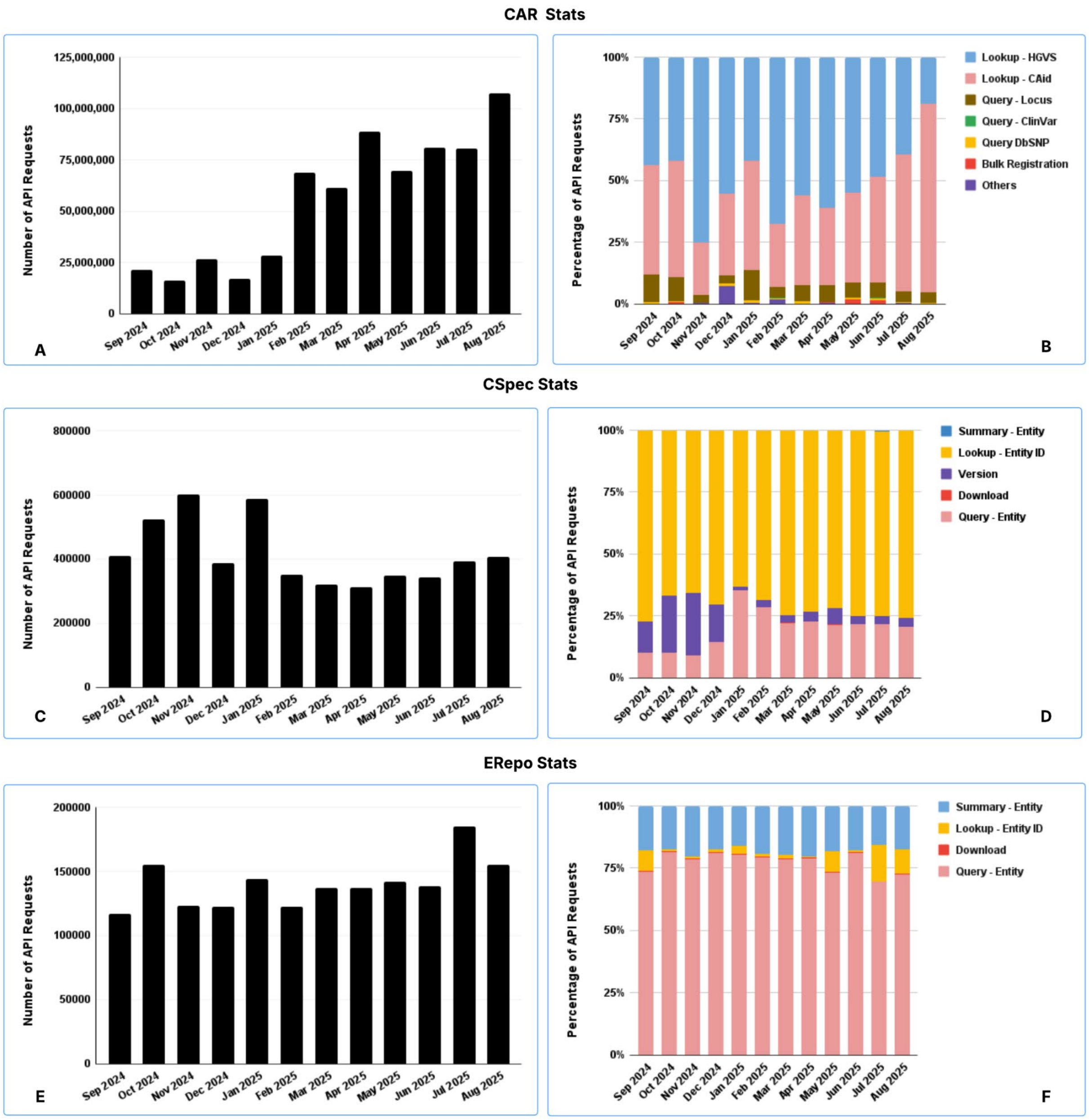

Figure S1: API Usage Statistics related to the section *ClinGen-developed FAIR variant data and knowledge are widely accessed via API endpoints* and Figure 1. Panel A: Total number of monthly CAR API requests. **Panel B**: Usage of 7 CAR API endpoints. “Others” includes API requests to query by ExAC, ClinVar RCV, MyVariantInfo, gene name, etc. **Panel C:** Total number of monthly CSpec API requests. **Panel D**: Usage of 5 CSpec API endpoints. **Panel E**: Total number of monthly ERepo API requests. **Panel F**: Usage of 4 ERepo API endpoints.

ID	Bridge2AI Criterion	Recommended Practice	API Microservices
			CAR, CSpec, ERepo, LDH
0	FAIRness		
0.a	Findable	Deposit datasets in a searchable FAIR-compliant data repository providing globally unique persistent identifiers resolvable to searchable, machine-readable, richly-descriptive metadata, including a link to the dataset if available.	CAR, CSpec, ERepo and LDH are FAIR-compliant data repositories.
0.b	Accessible	Descriptive metadata should always be available and accessible. Common access protocol.	CSpec, ERepo, LDH: Select entities only. Accessible via HTTP APIs.
0.c	Interoperable	Wherever possible, provide data and metadata using formally defined specifications for digital objects.	CAR, CSpec, ERepo and LDH entity data formatted according to formally defined specifications when they exist or machine-readable JSON.
0.d	Reusable	Attach a clear and accessible data usage license that allows the responsible use of AI/ML applications.	CAR, CSpec, ERepo and LDH are publicly accessible. Data usage terms in general are mentioned in the ClinGen website - https://www.clinicalgenome.org/docs/terms-of-use/. Data usage terms are also accessible through specific API endpoints as well. For example, note the LDH API endpoints for both the software license (https://ldh.genome.network/ldh/srvc/license) and the data terms of use (https://ldh.genome.network/ldh/srvc/license/tou).
1	Provenance		
1.a	Transparent	Identify sources of data.	CSpec & ERepo API payloads are linked to Variant Curation Expert Panels. LDH records all link back to the original data source.
1.b	Traceable	Identify important data transformation steps.	Criteria specification (CSpec record) and ERepo variant classifications are versioned. All the changes are traceable and interpretable.
1.c	Interpretable	Make software for key data transformation and analysis steps available in a sustainable repository.	Criteria specification (CSpec record) and ERepo variant classifications are versioned. All the changes are traceable and interpretable.
1.d	Key Actors Identified	Identify the people and organizations responsible for producing, obtaining and processing the data.	CSpec & Erepo API payloads are linked to Variant Curation Expert Panels. LDH

ID	Bridge2AI Criterion	Recommended Practice	API Microservices
			**CAR, CSpec, ERepo, LDH**
**0**	**FAIRness**		
0.a	Findable	Deposit datasets in a searchable FAIR-compliant data repository providing globally unique persistent identifiers resolvable to searchable, machine-readable, richly-descriptive metadata, including a link to the dataset if available.	CAR, CSpec, ERepo and LDH are FAIR-compliant data repositories.
			records link back to the data source.
**2**	**Characterization**		
2.a	Semantics	Use full descriptive metadata for datasets, including a detailed abstract, dataset keywords, and subject-specific vocabularies (e.g., MeSH for biomedical data) to enable detailed search and discovery.	CAR, CSpec, ERepo and LDH datasets are searchable using gene, disease MONDO Ids, etc.
2.b	Statistics	Provide appropriate statistical characterizations of key features of the dataset (e.g. demographics) where appropriate.	NA
2.c	Standards	Provide a machine-readable data dictionary or schema for each dataset, linked to the dataset metadata, and referencing any important applicable standards.	This is entity type specific for the LDH. Some entity records include links to schema. CAR, CSpec and ERepo APIs link to relevant context.
2.d	Potential Sources of Bias	Describe known sources of bias in the data and assumptions made in collecting, processing, or interpreting the data.	CSpec, ERepo and LDH contain variant curation data for a subset of ClinGen relevant genes and variants. Because ClinGen focuses on mendelian genetic disorders, clinically relevant genes and their variants in this category are likely over-represented compared to other genes and variants in these datasets. Additionally, genomics data historically over-represents individuals of European ancestry and that bias may be present in these datasets as well.
2.e	Data Quality	Have quality control procedures been applied? If so, provide a link to a description.	LDH: select entities only.
**3**	**Pre-model Explainability**		
3.a	Data Documentation Template	Machine-readable metadata and/or a linked human-readable document should support a domain-appropriate subset of the information in Datasheets.	NA

ID	Bridge2AI Criterion	Recommended Practice	API Microservices
			**CAR, CSpec, ERepo, LDH**
**0**	**FAIRness**		
0.a	Findable	Deposit datasets in a searchable FAIR-compliant data repository providing globally unique persistent identifiers resolvable to searchable, machine-readable, richly-descriptive metadata, including a link to the dataset if available.	CAR, CSpec, ERepo and LDH are FAIR-compliant data repositories.
3.b	Fit for Purpose	Identify appropriate and inappropriate use cases for a given data set in AI applications. Link to any previously published analyses using this data.	NA
3.c	Verifiable	Provide a mechanism for ensuring the integrity of each raw or processed dataset, such as a checksum.	Not supported.
**4**	**Ethics**		
4.a	Ethically Acquired	Describe ethical data acquisition consistent with accepted principles.	Data was submitted by data owners, or is publically available.
4.b	Ethically Managed	Data management, including processing, storage and access and use are expected to align with ethical principles throughout the health AI lifecycle.	CAR, CSpec, ERepo and LDH exclusively use publicly available data, and provide equal, transparent access to all users for scientific and other legitimate purposes.
4.c	Ethically Disseminated	Specify a licensing agreement and/or data use agreement on as open terms as ethical and sustainability considerations permit.	All data is publicly accessible.
4.d	Secure	Specify security requirements for storing and accessing this data, e.g. “public”, “controlled access only”, etc.	Public, except for CAR registration endpoints.
**5**	**Sustainability**		
5.a	Persistent	Ensure that unprocessed data is preserved in an archive adhering to privacy laws and retention guidelines, enabling future reprocessing and updated publishing of revised data.	NA
5.b	Domain-appropriate	Ensure single domain raw or processed data (as appropriate) is deposited in a FAIR domain-appropriate specialist repository if available.	CAR, CSpec, ERepo and LDH are FAIR data repositories under ClinGen governance.
5.c	Well-governed	Select a repository that facilitates how data will be stewarded in the future and governance that accounts for maintenance, terms and policy changes, and fairness.	CAR, CSpec, ERepo and LDH are FAIR data repositories under ClinGen governance.
5.d	Associated	Document project-level connections between data components and elements in a machine-readable	CAR, CSpec, ERepo and LDH are associated with multiple products within

ID	Bridge2AI Criterion	Recommended Practice	API Microservices
			CAR, CSpec, ERepo, LDH
0	FAIRness		
0.a	Findable	Deposit datasets in a searchable FAIR-compliant data repository providing globally unique persistent identifiers resolvable to searchable, machine-readable, richly-descriptive metadata, including a link to the dataset if available.	CAR, CSpec, ERepo and LDH are FAIR-compliant data repositories.
		manner.	and outside of ClinGen. Associations are made through URLs and machine-readable data.
6	Computability		
6.a	Standardized	Datasets follow established, documented standards and their adherence to standards may be validated deterministically.	CAR, CSpec, ERepo and LDH entities use standard formats when they exist or machine-readable JSON. ERepo and LDH entities are represented in standards such as SEPIO, GA4GH, etc.
6.b	Computationally Accessible	Provide a mechanism to access data either through established exchange protocols or a well-documented API.	CAR, CSpec, ERepo and LDH are well-documented RESTful APIs.
6.c	Portable	Maximize portability across computational resources where possible. If working with the data requires specific resources, provide machine-readable documentation defining these resources.	NA
6.d	Contextualized	Include any considerations regarding splits of the data, including any information withheld at any point of data collection and processing. If possible, provide examples of data components to facilitate understanding of their general structure and content.	NA

Table S1: AI-readiness evaluation for the resources listed in Table 1: CAR, CSpec, ERepo and LDH API microservices.
